# Form-Profiling of Optics Using the Geometry Measuring Machine and the M-48 CMM at NIST

**DOI:** 10.6028/jres.111.027

**Published:** 2006-10-01

**Authors:** Nadia Machkour-Deshayes, John Stoup, Z. Q. John Lu, Johannes Soons, Ulf Griesmann, Robert Polvani

**Affiliations:** National Institute of Standards and Technology, Gaithersburg, MD 20899 USA

**Keywords:** asphere and free form optics, form-profilometry, optical calibration metrology, surface metrology

## Abstract

We are developing an instrument, the Geometry Measuring Machine (GEMM), to measure the profile errors of aspheric and free form optical surfaces, with measurement uncertainties near 1 nm. Using GEMM, an optical profile is reconstructed from local curvatures of a surface, which are measured at points on the optic’s surface. We will describe a prototype version of GEMM, its repeatability with time, a measurements registry practice, and the calibration practice needed to make nanometer resolution comparisons with other instruments. Over three months, the repeatability of GEMM is 3 nm rms, and is based on the constancy of the measured profile of an elliptical mirror with a radius of curvature of about 83 m. As a demonstration of GEMM’s capabilities for curvature measurement, profiles of that same mirror were measured with GEMM and the NIST Moore M-48 coordinate measuring machine. Although the methods are far different, two reconstructed profiles differ by 22 nm peak-to-valley, or 6 nm rms. This comparability clearly demonstrates that with appropriate calibration, our prototype of the GEMM can measure complex-shaped optics.

## 1. Introduction

Aspheres and free-form optics are high performance optical products, but their use is limited by the difficulty and high costs of measuring their shapes. For spherical or flat surfaces, full-aperture interferometry combines fractional nanometer resolution with wide-ranging shape utility and is the preferred way of easily and precisely measuring optical form or profile. For aspheric and free form surfaces, full-aperture interferometry is neither simple nor inherently accurate. The problems with general interferometry are the instrument’s limited dynamic range and non-common path errors; as an example of the effects of the three problems, see Fig. 2. Engineering a reference wave front to closely match the test part’s true shape minimizes all three problems, but this means full-aperture interferometry requires a null optic. When spherical and flat optical surfaces are measured a calibration-grade sphere or flat serves as the reference. With aspheric and free form optics a null is expensive to make and needs special care in the use. Four recognized methods to measure aspheric optics are: full aperture interferometry with a computer generated hologram (CGH) as the null, sub-aperture interferometry using “stitching” to make a composite from many sub-aperture interferograms, which have small deviations to a sphere or flat [1]; the coordinate measuring machine (CMM), when mechanical probing of the surface is acceptable [2]; and often, a contacting or non-contacting long trace form profilometer for measuring either the part’s slope or profile [3].

## 2. Prototype Geometry Measuring Machine (GEMM)

Using reconstructions of geometry from curvature measurements, the developing GEMM instrument explores another alternative. Differential geometry offers curvature as a way to measure form or profile, and use of curvature has already had a brief history [4–17]. Curvature is an intrinsic geometric property of curves and surfaces, which means curvature can completely determine an object’s form regardless of its orientation relative to an external reference. This is an important help to nanometer level metrology of complex surfaces. Considering the simplest definition of curvature, the curvature of a circle or sphere is the reciprocal of the radius of the circle or sphere. For aspheric and free form optical metrology a rigorous definition is needed. Curvature can be defined, for a Cartesian coordinate system, using Eq. (1). This is a non-linear differential equation relating a one-dimensional profile *z*(*x*) to its curvature, *K*(*x*), for any value of *x* [18]:

$$\frac{d^{2}z(x)}{dx^{2}}=K(x){\left(1+{\left(\frac{dz(x)}{dx}\right)}^{2}\right)}^{3/2}.$$ (1)

Measuring curvature requires solving mathematical obstacles to ensure the accuracy of a reconstructed profile, but compensates with wide-ranging shape measuring versatility and reduced mechanical implementation problems. Considering the potential advantages of curvature and following the successes of the Physikalisch-Technische Bundesanstalt (PTB) with the Large Aperture Curvature Sensor (LACS) and other instruments [6–14], NIST committed itself to investigating the use of curvature to measure form.

**Fig. 1:**
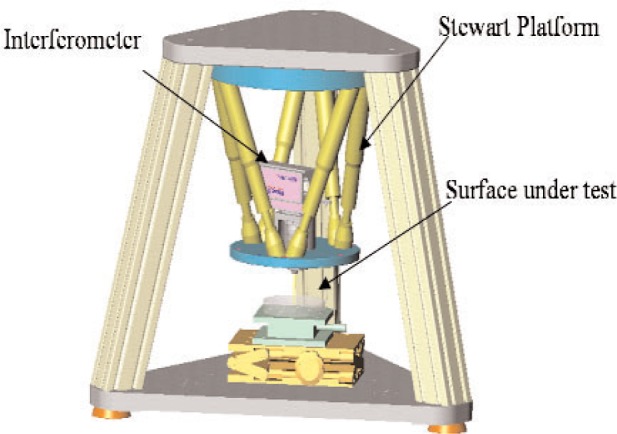
Schematic drawing of the NIST Geometry Measuring Machine

LACS and GEMM differ in design and primary use. The LACS uses a rotating air bearing supported arm to swing the curvature sensor in an arc over the optic. There is a wrist at the sensor mount, which orients the sensor normal to the optic’s surface, a requirement of the measurement. When the sensor is normal to the surface or the slope is zero, Eq. (1) simplifies to *K*(*x*) equals the second derivative of the surface form. GEMM uses a Stewart platform to position and orient the sensor. GEMM is to be a 3D shape-measuring instrument. Figure 1 shows GEMM schematically. The NIST instrument is installed within a temperature-controlled laboratory on a vibration-isolated granite table. It uses a commercial, miniature, Twyman-Green phase-measuring interferometer as curvature sensor. The Stewart platform workspace restricts the prototype to test parts with a maximum diameter of 110 mm. The sensor’s objective lens temporarily restricts use of the prototype GEMM to optics with > 4 m radius of curvature (ROC), or < 0. 25 m^−1^ curvature [17]. The sensor measures a local topography at many sites along a symmetry line on the part’s surface. Using the 512 sensor pixels lying on the center-line of the field of view, curvature extraction fits a circle to the site topography and assigns that curvature to the site.

To move the sensor along the scan line, and position it normal to the surface, GEMM uses a commercial Stewart platform. This is a stiff, stable motion system with six degrees of freedom, less than 2 µm positioning uncertainty for translation and less than 20 µrad angular positioning uncertainty for rotation, and a useful robotic intelligence [19]. The high-level language compatible robot has two setup uses. It helps register the GEMM coordinate axes to the part, and precisely aligns the scan line to the center of symmetry of the part. This alignment is of key importance when comparing profiles obtained from different instruments [17], and especially for establishing the correct position and orientation of the profile on the part. Setup starts with manually placing an optic onto a vertical stage, lifting it into the objective lens image plane, moving the optic within sensor view, and finally centering the sensor over three sample fiducial markings on the fixture. The robot remembers and uses these later to define the scan line and measurement sites. During a measurement, the robot has other duties. It moves the sensor along the scan line and aligns the sensor normal to the surface at each site. Finally, the sensor software—in process—does the image analysis, reducing the phase map at each site to a representative curvature, and storing the measurement data.

## 3. From Curvatures to a Profile

In Cartesian coordinates, a non-linear differential equation relates the one-dimensional profile *z*(*x*) to its curvature *K*(*x*) at each point x [18]:

$$\frac{d^{2}z(x)}{dx^{2}}=K(x){\left(1+{\left(\frac{dz(x)}{dx}\right)}^{2}\right)}^{3/2}.$$ (2)

When the curvature *K*(*x*) is measured, Eq. (1) must be solved to determine the profile. This can be accomplished using one of the standard methods for solving differential equations. Alternatively, an integration procedure described by Elster et al. [16] can be used to solve Eq. (1), which is now briefly reviewed.

$$\begin{array}{l} \text{Let}\;P(x)=z^{\prime }(x).\\ \text{Then}\;P^{\prime }(x)=K(x){\left[1+P{\left(x\right)}^{2}\right]}^{3/2}.\\ \text{Thus}\;\frac{P^{\prime }(x)}{{\left(1+P{\left(x\right)}^{2}\right)}^{3/2}}=K(x).\\ \text{So}\;\int \frac{dP}{{\left(1+P{\left(x\right)}^{2}\right)}^{3/2}}=\int K(x)dx,\\ \;\text{but}\;\int \frac{dP}{{\left(1+P{\left(x\right)}^{2}\right)}^{3/2}}=\frac{P(x)}{\sqrt{1+P{\left(x\right)}^{2}}}.\\ \text{Now let}\;\psi (x)=\int K(x)dx.\\ \text{We then have}\;\frac{P(x)}{\sqrt{1+P{\left(x\right)}^{2}}}=\psi (x).\\ \text{And finally}\;P(x)=\frac{\psi (x)}{\sqrt{1-\psi {\left(x\right)}^{2}}}=z^{\prime }(x). \end{array}$$ (3)

To summarize the process, the first derivative of the profile is expressed as a function of the integration of curvature, and the profile calculated with a simple integration of the first derivative function. Because the measured curvatures are a sequence of point measurements, numerical integration is applied to the curvature measurements to get the (*x*) function and the *z*(*x*) profile. For a numerical integration method, we use the cubic spline method followed by Simpson’s rule for integration. The unknown integration constants determine the position and orientation of the profile, but these are not relevant.

## 4. Description of the Test Mirror

**Fig. 2:**
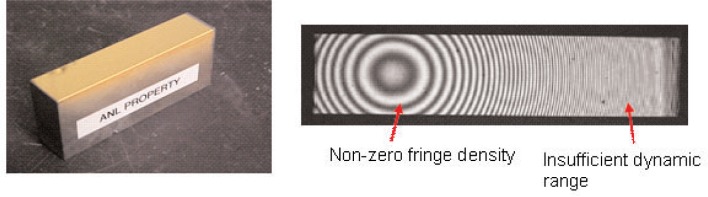
The APS #1 Mirror together with the corresponding full-aperture XCALIBIR interferogram. The interferogram has three deficiencies: the interferometer is not at null; the image—to right of center—has too many fringes, which means a spherical deviation beyond XCALIBIR’s working range; and last the ghost images indicate non-common path errors.

The test piece for this study is a free-form optic, an elliptical torus, and is one of two in a Kirkpatrick-Baez imaging system used in an x-ray beam-line at the Advanced Photon Source (APS) of the Argonne National Laboratory (ANL). A photo of the APS#1 mirror and an interferogram obtained using the eXtremely accurate CALIBration InterferometeR (XCALIBIR) at NIST, are shown in Fig. 2. XCALIBIR is a 300 mm aperture multi-configuration interferometer developed to calibrate flats and spheres for figure errors, spheres for radius of curvature, and more importantly to calibrate aspheres and free-form optics for figure. In all cases, the expectation is fractional nm accuracy. XCALIBIR is an extraordinarily precise instrument located in a very stable environment. To use XCALI-BIR with aspheres and free-forms, both stitching and CGH methodologies are used. However, the interferogram shown in Fig. 2 was made full-aperture, using a flat as the reference, and without a null condition. The intention is to show the difficulties of measuring even this weak free-form optic without an appropriate null, or using stitching or CGH methods as the correction.

The silicon substrate is a 90 mm long, 19 mm wide, and 20 mm tall block. The reflective top surface of the block was polished to approximately 84 m radius of curvature. To give the mirror the elliptical profile, a gold coating of varying thickness was deposited onto a spherical base. The shape varies from nearly spherical at one end to elliptical at the other. Because the “true” elliptical profile of this mirror is unknown, we chose to compare the profile measured with GEMM to the profile measured with the NIST Moore M-48 CMM.

## 5. Moore M-48^1^ CMM

**Fig. 3:**
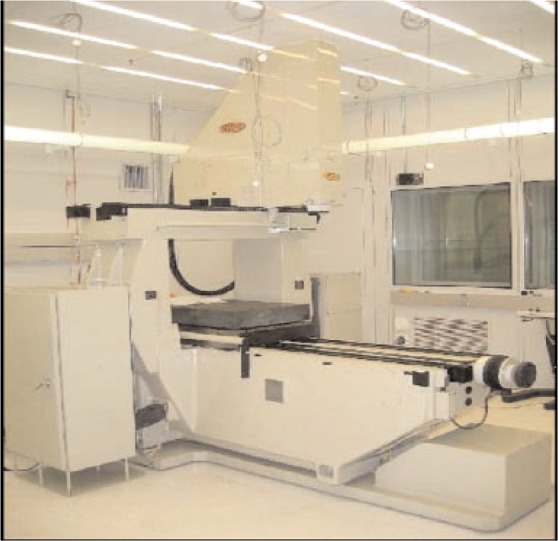
Photograph of the Moore M-48 Coordinate Measuring Machine at NIST^1^

The M-48 coordinate measuring machine (CMM) is shown in Fig. 3, and is one of the most accurate CMMs—for its size—in the world. The machine structure consists of a heavy cast iron, jig-grinder base set on three vibration-damping mounts. The X-axis table and the Y-axis cross-carriage motions are carried out by high-precision lead screws immersed in oil baths, which are guided by precision double-“V” roller ways, and assisted by constant force springs to reduce backlash to insignificant levels. The Z-axis motion is achieved through a counterweighted ceramic ram hung from another precision lead screw and guided by air bearings and constant force springs. Laser interferometers are used on all three axes.

A 200 mm thick, kinematically-mounted granite surface plate on the machine table transforms complex table bending errors into more easily corrected rigid body motion errors. The machine is housed in a very stable laboratory environment. The room is maintained at 50 % humidity and 20 +/− 0.01 °C degrees Centigrade. For still finer assessment of the thermal environment, the local machine temperature is monitored with 14 sensors placed in and around the machine. The probing system uses hydraulically damped and independently deformable parallelograms for all three axes of motion and provides repeatability at the level of 10 nm. The vertical or *z* axis resolution is 10 nm. Redundant error mapping and process control techniques achieve 2D positioning accuracy over an area of 600 mm × 600 mm of better than 50 nm for optical and touch probe measurements.

## 6. Measurement Details

Previously, the Argonne National Laboratory/APS Long Trace Profiler, an optical non-contacting profilometer, NIST XCALIBIR, Moore M-48 and GEMM, were all used to profile the APS#1 mirror. The profiles agreed within 20 nm peak-to-valley (P-V), but the average radius of curvature of the optic could not be reported with high precision [17]. The problem was the measurements were not registered to a common coordinate axes for the four measurement traces, nor were the sites for the curvature values exactly the same. This meant the profiles had unknown lateral shifts, resulting in unknown relative biases in the average radius. This time we made the comparison using two improvements: forced registry of the coordinate axes, which meant the measurements were made at the same sites along the same symmetry line, and more importantly an integral calibration of the curvature sensor was used to improve the curvature value accuracy.

### 6.1 Measurement Uncertainty

**Fig. 4:**
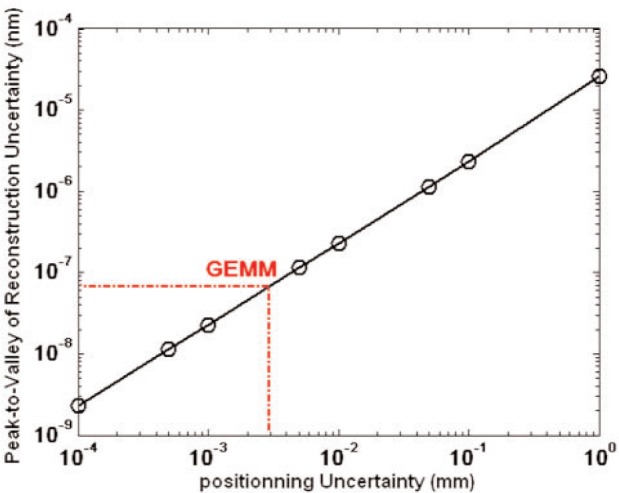
The reconstruction error is shown as function of sensor position uncertainty

Using Monte Carlo simulations, the uncertainty associated with GEMM metrology was extensively analyzed [17]. Profiling is sensitive to two types of uncertainty: sensor positioning error and curvature estimation. Sensor positioning error is a mix of two error sources. One is poor registry of the part within GEMM and another instrument, because different scan lines were used. The other is the GEMM robot inaccurately positions the sensor at the sites or the robot does not orient the sensor normal to the surface at the sites. We have already considered the specific effects for the APS#1 mirror and GEMM 10 mm objective. Past practice was greater than 150 µm coordinate axes registry error, but current practice results in a mismatch of less than 20 µm between GEMM and the Moore M-48. This is mainly due to the pixel resolution of GEMM’s interferometer. This should cause an estimated 8 nm (P-V) profile deviation for APS#1. Giving the robot full control reduces site-positioning errors to within 2 µm, and sensor orientation errors to within 20 µrad [19]; so the estimated reconstruction profile uncertainty is fractions of a nanometer for APS#1; see Fig. 4. Separation error and random noise, which bias the curvature sensing and extracted value, respectively, are two other large error sources. Separation error or bias in the curvature sensor causes a circular error in reconstructed profiles. As shown in a later section, calibration of the curvature sensor significantly reduces separation error. Noise is an end product of the irreproducibility of the curvature extraction process. Related to the interferometer’s resolution, the 4 nm random error of the 10 mm objective lens, is low enough for reconstructions of less than a 10 nm (PV) uncertainty with 10 m or larger radius parts [17].

### 6.2 Test Part Coordinates

**Fig. 5:**
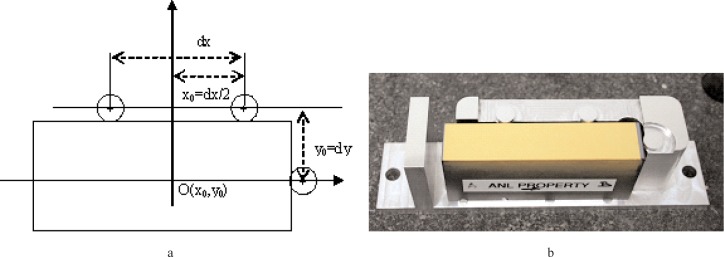
(a) A schematic showing how the fixture aids defining the coordinate system, and (b) The APS #1 mirror placed in the fixture and ready for profiling

To compare the profiles measured here, GEMM and the Moore M-48 were forced to use the same part coordinate axis system through a simple sample-mounting fixture. The fixture allows both instruments to measure the part profile in the same place. To define the coordinate system, the fixture has three circular posts with 8 mm diameter, which define the *x* and *y* axes of the coordinate system, and make point contact with the test part; see Fig. 5. At the center, each post has a 125 µm diameter hole. The CMM probes the post perimeters to define their centers and align the coordinate axes. The GEMM operator uses the interferometer to image the three holes, and the robot defines the coordinate axes for the measurements, and in this way the M-48 and GEMM become registered.

### 6.3 Sensor Calibration

GEMM uses a two-step measurement procedure. First the sensor is calibrated using three spherical artifacts of known curvature, and second the optic is profiled. The need to calibrate the interferometer each time is easily explained. Small errors arise from residual power in the sensor’s reference flat. The largest error results from small laboratory environmental fluctuations. Likely, the fluctuations affect the objective lens assembly geometry, which then has a strong and spurious effect on the separation value or bias of the curvature sensor. Because double integration is used to obtain the profile from curvature, a biased curvature sensor yields a circular error in the reconstructed profile, and the peak-to-valley value of that error increases rapidly with part diameter. The simplest relationship between the true curvature, *K*, of a test surface, and the measured value, *K_m_*, is a polynomial function:

$$K=\alpha +\beta \cdot K_{m}+\gamma \cdot K_{m}^{2}\ldots$$ (4)

To obtain the needed profile accuracy, the calibration coefficients *α*, *β*, *γ*, … are determined as an integral part of the profile measurement. To calibrate GEMM, five spherical artifacts are available and range from the largest 0.125 m^−1^ curvature, down to zero curvature, a flat.

**Fig. 6:**
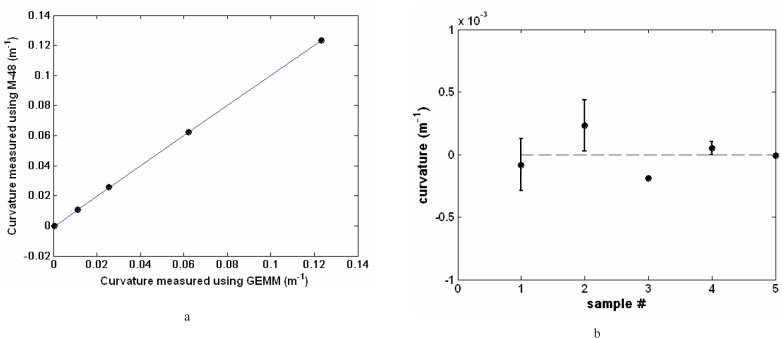
(a) The M-48 CMM-GEMM calibration curve was made using the 0.125 m^−1^, 0.05 m^−1^, 0.020 m^−1^, and 0.01 m^−1^ curvature and flat artifacts. The best-fit line is indicated; and (b) the same data with the best-fit line subtracted to show the residuals for each of the artifacts. The error bars in this figure indicate the standard uncertainty for three of the artifacts.

To initially assign a true curvature value, *K*, for use with Eq. (4), and to better understand the details of the curvature calibration of GEMM, all five were profiled with the Moore M-48, and Fig. 6a plots *K* (CMM) versus *K_m_* (GEMM). The curve in Fig. 6a is a best-fit line to the five CMM evaluations, with a slope of 1.022. Although a power function could be used, the linear approximation of Eq. (4) yields a sufficiently good description of the sensor bias, and simplifies the use. Figure 6b shows the deviations of the five M-48 CMM true curvature values to the best-fit line. Repeated measurements were made with three of the artifacts; so standard deviations are available for only three, and these are shown in Fig. 6b. Additionally the nominal, best-fit line, and M-48 CMM true curvature values are numerically summarized in Table 1. Because the artifacts are less than perfect spheres, forced registry of the sample fixture in the two instruments was necessary and assured the accuracy of the definition of the overall calibration of GEMM.

Calibration is the first step in profiling a test part, and as a first step uses only three of the artifacts to update the coefficients, *α*, *β*, of Eq. (4). The flat is always used, because it defines the offset or sensor bias, and the test part’s nominal curvature determines the choice of the others. The selection has the test optic lying somewhere between the artifact pair. Repeated 3-artifact-calibrations with GEMM show the offset coefficient changes markedly over several days, but the slope of the line is essentially constant. So bracketing the test piece with two artifacts coupled with interpolation enhances the precision of the GEMM curvature definition.

### 6.4 GEMM Long Time Repeatability

**Fig. 7:**
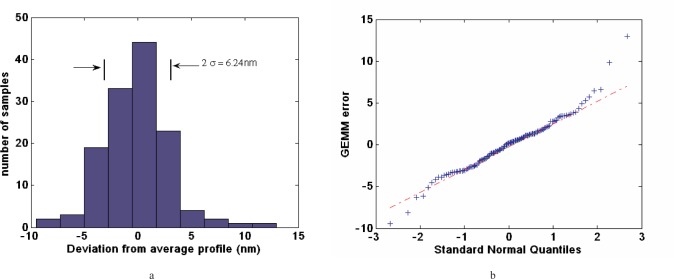
GEMM repeatability (a) a histogram showing the measured error distribution, and (b) GEMM error against the Standard Normal Quantiles to test for a normally distributed error.

Starting early-December 2005 and running throughout February 2006, a series of four profiles of APS #1 were measured to estimate the repeatability of GEMM. Our measure of repeatability is the deviation of the individual profiles with respect to their overall averages and the individual deviations. The distribution of the repeatability error of the four profiles is plotted in Fig. 7a. The one standard deviation of the repeatability is 3.12 nm. As shown in detail in Fig. 7b the error is almost normally distributed.

## 7. Measurement of the APS #1 Mirror

The Moore M-48 and GEMM measured the elliptical mirror four times each to determine whether the two instruments would obtain the same profile. The test strategy was for the CMM to define for both instruments: the common coordinate axes to use; with respect to the width of the mirror, the mid-span location of the scan line to use; and the 33 sites for the curvature measurements each spaced 2 mm apart. The maximum error in the relative position of the profiles is estimated to be smaller than 20 µm, and is mainly due to not exactly registering the small three sample fiducials in GEMM. The two average profiles are plotted in Fig. 8a; which covers the full scan trace, or thirty-three measurement sites.

### 7.1 Analysis of the Profiles

With respect to a common best-fit fourth order polynomial through all profiles, Fig. 8b shows the average deviation and replication standard deviation for both the GEMM and M-48 profiles. The full set of 33 sites is reported, and the maximum difference between the GEMM and the CMM profiles is 22 nm (P-V). Considering Figs. 8a and 8b, the two profiles seem indistinguishable from one another and so the question becomes are they statistically indistinguishable? Whether the two profiles are indistinguishable and the statistical confidence level was tested using variations of the Student’s t-test and two ways of viewing the 33 measurements.

Student’s t-test uses the null hypothesis, standard error of the mean and a sample size weighted-lumped standard deviation to learn the statistical significance, at a confidence level, for the difference in two means. Importantly, the traditional Student’s t-Test assumes the variances of the two means are roughly equal. Considering Fig. 8b, the GEMM and M-48 variances are different and often the M-48 values are larger. But we will start answering the statistically evaluated comparability question, using traditional Student’s t-test. Later in the Appendix, we revisit the comparability question, but with far more care and statistical elegance. There we take into account the variances are different, and more importantly our consideration of the 33 measurement pairs as a set. Addressing unequal variances and 33 measurements as one, requires solving a Behrens-Fisher problem [23], and just how we do that is detailed in the Appendix. Either way the same conclusion results; the GEMM and CMM profiles are statistically indistinguishable.

**Fig. 9:**
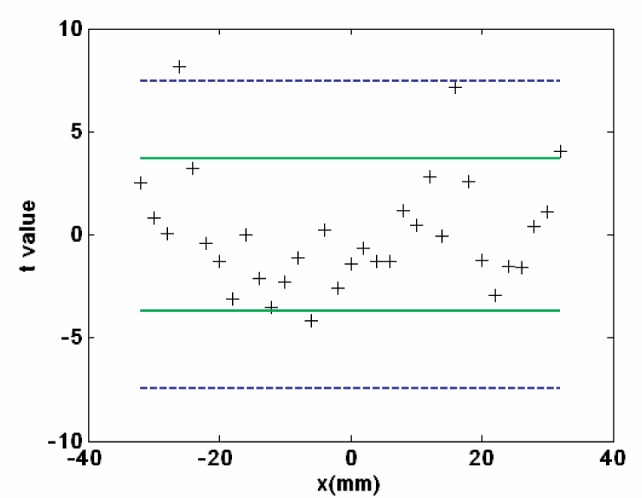
The 33 t-values considered either independently or as set. The upper-lower green boundaries define the 99 % confidence interval for individual site differences, and the blue boundaries define the 99 % confidence interval when the 33 values set is considered.

To compare the profiles with Student’s t-test, we chose to use the site values for our analysis rather than comparing two best-fit curves to the GEMM and CMM data. The choice was deliberate. Given the large radius of curvature of APS #1 we expected the best-fit curves would minimize registry and positioning errors, and that would be inappropriate. The measurement sites were tested for failure to meet the null hypothesis in two ways: Consider the profiles as a series of 33 fully independent mean differences, and second consider the data as a single set with 33 values, which is the less stringent test. Figure 9 shows the results of using the t-Test in the two ways. In Fig. 9, the t-values are calculated for each mean difference and then plotted against the site location. The upper-lower green boundaries define the 99 % confidence interval for individual site differences, and the blue boundaries define the 99 % confidence interval when the 33 value set is considered. As expected, individual sites require a lower t-value (< 3), or the GEMM and CMM mean need to be very little different for “indistinguishable.” But there are four outliers.

When the data are considered as a set then t need only be < 7, and with one outlier. The difference in the t-values is expected, and is an end product of the multiplicity effect. This is both explained in detail and properly addressed in the Appendix. Notably, considered individually with Student’s t-Test or as a set, and with the care described in the Appendix, the two are “indistinguishable” at the 99 % confidence level.

## 8. Summary and Conclusions

The repeatability of GEMM was evaluated over three months and is 3 nm rms. The cause of this uncertainty is mainly laboratory environmental changes and—to a lesser extent—interferometer resolution. Profiling < 0.25 m^−1^ curvature or > 4 m radius of curvature optics is a temporary limitation of this prototype of GEMM. The Twyman Green sensor requires GEMM hold an exact separation distance between sensor and surface as LACS can, or measure this distance and correct the curvature value. A new NIST-designed sensor, the Small Aperture Digital Interferometer (SADI), is under construction and will expand the working range of curvatures. SADI will use a single 5 mm objective lens, is a Fizeau design, has a simplified optical assembly to reduce random noise, and needs two common path interferometers to enable simultaneous form and distance measurements.

Using this prototype of GEMM and the Moore M-48 at NIST, the profile of an elliptical mirror was measured with a difference of 21 nm (P-V). Using Student’s t-tests, the null hypothesis is found to be true; at all measured sites, the two profiles are the same. The comparability is attributed to two recent improvements of GEMM. One is calibrating GEMM for curvature as a preliminary to determining the profile. The other is achieving better measurement registry between GEMM and the M-48. A sample holding fixture and fully utilizing the robot’s capabilities ensures registry of the GEMM and M-48 coordinate axes a key requirement for comparison. Better registry reduced the positioning uncertainty of the part from more than 150 µm with no fixture to less than 20 µm with the fixture.

The GEMM prototype and M-48 reporting the same profile for APS#1 is significant. First, the comparability shows calibration, and especially calibration as a step in the measurement process, allows GEMM to measure a complex shaped optic to the same accuracy as the Moore M-48 CMM; and more importantly—in principle—curvature measurements now show promise for the calibration of complex optical surfaces.

## Figures and Tables

**Fig. 8a:**
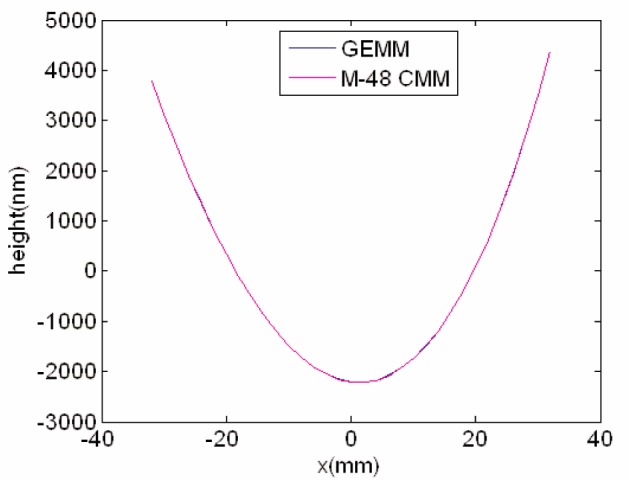
The GEMM and the M-48 CMM average profiles for the APS #1.

**Fig. 8b:**
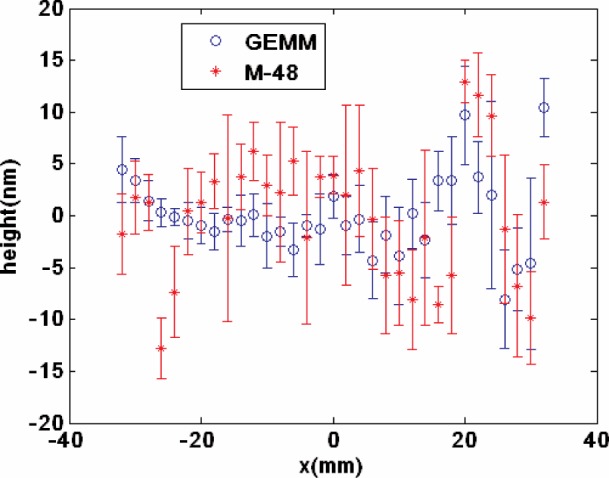
Deviation from a common best-fit fourth order polynomial of the CMM and GEMM profiles against the measurement sites The error bars are 1 standard deviation for the GEMM and M-48 measurements at each site.

**Fig. 10:**
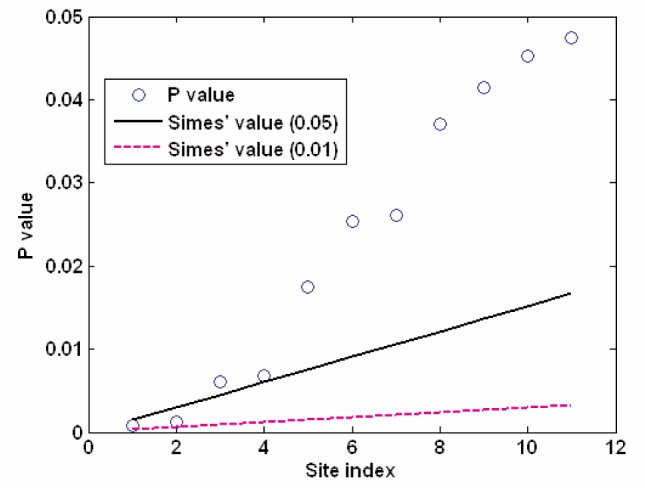
The results for the eleven sites with lowest P-values of testing hypotheses

**Table 1:** A Comparison of the Nominal, Best-Fit Line, and M-48 CMM estimate of the true curvature of the five artifacts. (Curvature × m^−1^)

Nominal Value	0.00	0.010000	0.020000	0.050000	0.125000
Line Best Fit Value	−0.0003237	0.01080734	0.02542557	0.06253948	0.12354971
Moore M-48 Value	−0.0002415	0.0105773	0.02561730	0.06248850	0.01235563

**Table 2:** The results (for the eight sites with lowest P-values) of testing multiple (33) hypotheses based on Simes’ modified Bonferroni procedure using the approximate t-distribution based on Satterthwaite formula for degree of freedom

Sites	25	4	14	33	11	28	8	23
Approx. dof	5	4.2	5.7	5.7	5.5	5.9	5.3	5.3
P value	0.0008	0.0012	0.0061	0.0068	0.0174	0.0254	0.0261	0.0371
Simes’ cutoff point (at 0.05)	0.0015	0.0030	0.0045	0.0061	0.0076	0.0091	0.0106	0.0121
Simes’ cutoff point (at 0.01)	0.0003	0.0006	0.0009	0.0012	0.0015	0.0018	0.0021	0.0024

## Footnotes

1 Certain commercial equipment, instruments, or materials are identified in this document. Such identification does not imply recommendation or endorsement by the National Institute of Standards and Technology, nor does it imply that the products are identified are necessarily the best available for the purpose.

## 9. Appendix

### 9.1 Student t-Test with Unequal Variances

The data populations are four profile measurements each, which were made with the M-48 CMM and GEMM. To describe the statistical methods, we denote the M-48 CMM profiles by *f*_1_*_i_*(*x*) and GEMM profiles by *f*_2_*_i_*(*x*), where *i* = 1, 2, 3, 4 and *x* takes on one of the 33 measured locations. The mean profiles from the two instruments are given by:

$${\bar{f}}_{1}(x)=\frac{1}{4}\sum_{i}f_{1i}(x),\;{\bar{f}}_{2}(x)=\frac{1}{4}\sum_{i}f_{2i}(x).$$

The standard deviations from the repeated measurements at a given location are given by

$$\begin{array}{l} s_{1}(x)=\sqrt{\frac{1}{3}\sum_{i}{\left(f_{1i}(x)-{\bar{f}}_{1}(x)\right)}^{2}},\\ s_{2}(x)=\sqrt{\frac{1}{3}\sum_{i}{\left(f_{2i}(x)-{\bar{f}}_{2}(x)\right)}^{2}}. \end{array}$$

Thus, the combined standard error for the difference ${\bar{f}}_{1}(x)-{\bar{f}}_{2}(x)$ is given by

$$s_{c}(x)=\sqrt{\frac{1}{4}s_{1}^{2}(x)+\frac{1}{4}s_{2}^{2}(x)}.$$ (5)

At a given location *x*, the test statistic for comparing two mean measurements is based on the t-statistics (ratios):

$$t(x)=\frac{{\bar{f}}_{1}(x)-{\bar{f}}_{2}(x)}{s_{c}(x)}.$$ (6)

Accepting the null hypothesis that the two mean profiles are the same, the t-statistics should follow the standard t-distribution with six degrees of freedom.

At any given location *x*, the null hypothesis of *f*_1_(*x*) = *f*_2_(*x*) is rejected if

$$|t(x)|\geq F_{t_{6}}^{-1}(1-\alpha /2),$$ (7)

where α is the pre-chosen level of significance. Here $F_{t_{6}}^{-1}(q)$ denotes the inverse of the cumulative distribution function at probability *q*, which is the *q*th quantile of the standard t-distribution with six degrees of freedom. In addition, at a given location *x*, a point wise (1 − α) × 100 % confidence interval for *f*_1_(*x*) − *f*_2_(*x*) is provided by:

$${\bar{f}}_{1}(x)-{\bar{f}}_{2}(x)\pm \xi_{1-\alpha /2}s_{c}(x),\;\text{where}\;\xi_{1-\alpha /2}=F_{t_{6}}^{-1}(1-\alpha /2).$$ (8)

Equivalent to the rule provided by (7), we can reject the null hypothesis of *f*_1_(*x*) 2212 *f*_2_(*x*) = 0 at the significance level α if 0 is not contained in the interval provided by (8). Thus, rules (7) and (8) can be used interchangeably for hypothesis testing.

We need to point out two cautions in the use of the t-test based comparison procedure described above. First, the standard t-test procedure for two-sample is based on the equal variance assumption and uses a combined variance estimator (cf. Ch. 6, [20]). Since the variance for the M-48 CMM is typically much larger than the GEMM measurements, the equal variance assumption is obviously violated. Strictly speaking, the inequality of variances at some locations means we should use the Behrens-Fisher distribution [23] in (7) and (8). Because the Behrens-Fisher distribution depends on the unknown ratio of variances, using it is quite involved and requires special tables. A simple approach is to use the Satterthwaite formula to give the approximate degree of freedom (dof) for using the standard t-distribution as described in Ch 6 of [20]. Table 2 shows the results of the first eight smallest P-value calculations based on the Satterthwaite approximation. There are 11 sites with P-value<0.05, and 4 sites with P-value<0.01. However, this does not guarantee that the profiles at these sites are truly significantly different. This means that pure chance from measurement errors alone can produce these spurious small P-values. This is due to the effect of multiplicity when there are many (33) hypotheses being considered at the same time. In next section, we discuss how to adjust for the multiplicity effect so as to reduce the number of false positives.

### 9.2 Adjustment of Multiplicity of Testing at Many Sites

If more than one site is tested at the same time, the probabilistic statements in (7) and (8) are not correct. If there are many sites to be considered at the same time, the number of false positives due to using (7) and (8) can be substantial. For example, if all 33 sites are considered as a whole, the type I error can be as high as 33 × α, and the number of expected false positives is 33α. Accounting for the *multiplicity effect* is important, when the number of hypotheses being considered is large. On the other hand, one should be careful of over-adjusting since a very large threshold can lead to accepting anything, and lead to higher type I error, that of failing to detect a significant difference in the alternative [21]. Correctly setting the appropriate threshold for multiple testing can be tricky and is still active research issue in Statistics. Significant complication arises when there is potential *dependency* among the spatially contiguous sites. If dependence is ignored, one can apply standard multiple test procedure such as the Bonferroni or Simes’ modified Bonferroni test [22] based on ordered P-values. For testing a given number of hypotheses, say *m*=33, if any P-value of a given hypothesis is less than *α*/*m*, the joint null hypothesis is rejected at level of significance *α*, according to the Bonferroni procedure. However, usually the Bonferroni procedure gives a too small cutoff threshold. Simes [22] proposed an improved procedure: order the P-values according to *P*_(1)_ ≤ *P*_(2)_ ≤ … ≤ *P*_(_*_m_*_)_, then the joint null hypothesis is rejected if *P*_(_*_j_*_)_ ≤ *jα*/*m* for any *j* = 1, 2, …, *m*. Simes’ test is less stringent and so will detect more alternatives, and this is especially true when there are multiple hypotheses being rejected.

Table 2 shows for the first eight sites with the lowest P-values based on the Satterthwaite’s approximate dof’s for t-distribution approximation (for the 11 sites with P-values<0.05 see Fig. 10), the corresponding p-values, and Simes’ cutoff values at *α* = 0.05 and *α* = 0.01.

At *α* = 0.05, there are two significance sites according to both the Bonferroni procedure and the Simes’s procedure. At *α* = 0.01, there is no significant difference based on either the Simes’ test or the Bonferroni test. Notice that, *α* indicates the overall type I error of the multiple tests—a smaller *α* implies that the test has a smaller type I error, but has a larger type II error, or a lower probability of detecting the alternatives, thus explaining why we found two significance sites at *α* = 0.05 but failed to find any significance site at *α* = 0.01.
